# Retraction: A parallel randomized controlled trial examining the effects of rhythmic sensory stimulation on fibromyalgia symptoms

**DOI:** 10.1371/journal.pone.0230395

**Published:** 2020-03-06

**Authors:** The PLOS ONE Editors

After this article [1] was published, concerns were raised about aspects of the study design and conclusions that prompted the *PLOS ONE* Editors to re-evaluate the article, together with a member of our Editorial Board.

This study aimed to (1) evaluate whether the rhythmic sensory stimulation (RSS) treatments with different parameters would yield different effects on participants; and (2) evaluate the efficacy of RSS in reducing the severity of fibromyalgia symptoms. The reported experiments involved two RSS treatment groups, as described in the Methods, both of which received RSS treatment albeit with different specifications. The study did not include a true sham control treatment. The results of pre- versus post-treatment analyses reported in the article suggested that RSS stimulation may impact fibromyalgia symptoms. However, given the lack of a sham control group, one cannot distinguish effects of either treatment from placebo effects as would be needed to address the second study aim.

The lack of a placebo group was raised during pre-publication peer review and was acknowledged as a limitation in the article’s Discussion. However, after further consideration and post-publication input from a member of our Editorial Board, we concluded that the claims made in the article are not adequately supported by the results given the lack of the control group and the implications of this issue for the interpretation of the data. Therefore, the article does not meet *PLOS ONE*’s publication criteria, and so the *PLOS ONE* Editors retract this article. We regret that this issue in the study design was not fully addressed during the pre-publication peer review process.

TBJ and LRB did not agree with the retraction. DP, LP, and AG either did not respond directly or could not be reached.

At the time of retraction, the article [1] was republished to remove S1 Figure due to unresolved questions about copyright ownership.
